# Supplementary material for: Anatomical Tool as Additional Approach for Identifying Pharmaceutically Important Ephedra Species (Ephedraceae) at Gender Identity Level in Egypt
Source: Biology (Basel). 2024 Nov 18;13(11):947. doi: 10.3390/biology13110947 (PMC11591873; doi:10.3390/biology13110947)
Supplement: Supplementary file 1 [file biology-13-00947-s001.zip › biology-3277052-supplementary.pdf]

Table S1. Anatomical characteristics of the studied stems of *Ephedra* species/genders.

Species		Outline	Dermal tissues								Cortex				
			Cuticle	Epidermis		Papilla		Trichomes		The mean number of sunken stomata	Arrangement of chlorenchymatous tissues	Fibrous tissues			Arrangement of parenchymatous tissues
				Elongation	Composition	Presence	Mean number per section	Presence	Type			Arrangement	Mean number of patches of fibers	Lignification of patches of fibers	
1	♀	Terete	Thick	Tangentially -radially	Uniseriate	Present	200	Absent	Absent	45	Ill-defined	Ill-defined	Ill-defined	Ill-defined	Ill-defined
	♂	Ridged and furrowed	//	//	//	//	150	//	Absent	70	3-4 rows	2-3 rows	45	Lignified	1-2 rows (Polyhedral parenchyma)
2	♀	Terete	//	Tangentially	//	Absent	Absent	Present	Non-glandular, unicellular, uniseriate	75	1-2 rows	One row	55	Non-lignified	//
	♂	Ridged and furrowed	//	//	Biseriate	Present	140	Absent	Absent	60	2-3 rows	2-3 rows	110	//	4-5 rows (Polyhedral parenchyma)
3	♀	Terete	//	Radially	Uniseriate	Absent	Absent	Present	Non-glandular, unicellular, uniseriate	40	1-2 rows	1-2 rows	50	//	3-4 rows (Polyhedral parenchyma)
	♂	//	//	//	//	//	//	//	Non-glandular, unicellular, uniseriate	60	2-3 rows	//	90	//	2-3 rows (Polyhedral parenchyma)
4	♀	//	//	Radially -tangentially	//	Present	60	Absent	Absent	40	1-2 rows	//	80	//	1-2 rows (Polyhedral parenchyma)
	♂	//	//	Radially	//	//	50	//	//	30	3-4 rows	3-4 rows	150	Non-lignified and lignified	2-3 rows (Polyhedral parenchyma)
5	♀	//	//	Tangentially	//	//	40	//	//	45	//	//	100	//	1-2 rows (Polyhedral parenchyma)
	♂	Terete - ridged and furrowed	//	//	//	//	80	//	//	20	//	2-3 rows	120	//	2-3 rows (Polyhedral parenchyma)

Table S1. Cont.

Species		Endodermis	Continuity of the phloem fibers	Vascular tissues					Pith			Tanniniferous idioblasts		Stone Cells	
				Number of protoxylem arches	Secondary growth			Annual rings	Components	Width	Cavity	Presence	Occurrence	Presence	Occurrence
					Fascicular region		Components of interfascicular region								
					No of Tracheids arches/ (mean)	Tracheid width									
1	♀	Ill-defined	Patches	10	4	Narrow	Secondary phloem, lignified conjunctive parenchyma, and some tracheids	Absent	Lignified parenchyma & normal parenchyma	Wide	Absent	Present	Cortex & pith (abundant)	Present	Interfascicular region, end of protoxylem poles and pith
	♂	Present	//	//	5	Moderate	Secondary xylem / secondary phloem/tracheids	Present	//	//	//	//	Pith (abundant)	//	//
2	♀	Absent	Continuous ring	15	9	Wide	//	Absent	//	Narrow	//	Absent	Absent	//	End of protoxylem poles and pith
	♂	Present	//	//	7	//	//	//	Normal parenchyma	Wide	Present	//	//	//	Interfascicular region and end of protoxylem poles
3	♀	//	//	15-17	7	//	//	//	Lignified parenchyma & normal parenchyma	Narrow	Absent	//	//	//	//
	♂	Absent	//	//	8	//	//	Present	Normal parenchyma	//	//	//	//	//	//
4	♀	//	//	15	6	Moderate	//	Absent	//	//	//	//	//	//	//
	♂	Present	Patches	10	4	Narrow	Secondary phloem, lignified conjunctive parenchyma, and some tracheids	//	//	Wide	//	//	//	//	Interfascicular region
5	♀	Absent	//	//	3	//	//	//	//	//	//	//	//	Absent	Absent
	♂	Present	//	10-12	4	//	//	//	//	//	//	//	//	Present	Interfascicular region and end of protoxylem poles

Table S2. Stem epidermal characteristics of the studied stems of *Ephedra* species/genders.

Species		Epidermal cell characteristics				Stomatal characteristics						Trichomes presence	Crystals	
		Epidermal cell shape	Epidermal cell size		Anticlinal cell pattern	Stomatal type		Stomatal size		Stomatal index (%)	Stomatal length: width		Type	Abundance
			Length (µm)	Width (µm)		Pattern	composition	Length (µm)	Width (µm)					
1	♀	Short hexagonal	4.42 ± 0.33 **a**	2.61 ± 0.09 **abcd**	Smooth and straight	Pseudo-stripped	Anomocytic	2.29 ± 0.15 **ab**	1.04 ± 0.03 **b**	10.20	2.20	Absent	Solitary	Few
	♂	//	5.26 ± 0.35 **ab**	5.17 ± 0.19 **e**	//	//	Brachyparatetracytic	3.61 ± 0.30 **c**	2.63 ± 0.15 **d**	7.09	1.37	//	//	//
2	♀	Long hexagonal	5.42 ± 0.52 **ab**	2.45 ± 0.04 **abc**	//	Stripped	Anomocytic	1.74 ± 0.14 **a**	0.52 ± 0.06 **a**	15.12	3.34	Present	Druses	//
	♂	//	9.30 ± 1.11 **bc**	2.79 ± 0.10 **bcd**	//	//	//	3.14 ± 0.21 **bc**	0.90 ± 0.19 **ab**	12.28	3.48	Absent	//	//
3	♀	//	6.91 ± 1.85 **abc**	3.36 ± 0.25 **d**	//	//	//	2.27 ± 0.18 **ab**	0.82 ± 0.08 **ab**	14.13	2.76	Present	//	Abundant
	♂	Short hexagonal	6.56 ± 0.70 **abc**	3.32 ± 0.35 **cd**	//	//	//	2.39 ± 0.07 **ab**	0.94 ± 0.05 **ab**	11.04	2.54	//	//	//
4	♀	//	6.55 ± 0.87 **abc**	3.48 ± 0.23 **d**	//	//	//	2.70 ± 0.50 **abc**	1.97 ± 0.02 **c**	2.17	1.37	Absent	//	Few
	♂	Long hexagonal	10.94 ± 0.38 **c**	1.71 ± 0.21 **a**	//	//	//	3.09 ± 0.02 **bc**	0.86 ± 0.21 **ab**	4.42	3.59	//	//	//
5	♀	//	7.28 ± 0.68 **abc**	2.28 ± 0.19 **ab**	//	//	//	2.49 ± 0.04 **ab**	0.67 ± 0.34 **ab**	5.50	3.71	//	//	Abundant
	♂	//	5.43 ± 0.85 **ab**	2.38 ± 0.43 **ab**	//	//	//	2.64 ± 0.21 **abc**	0.95 ± 0.67 **ab**	18.05	2.77	//	//	Few

1. *E. alata;* 2. *E. aphylla;* 3. *E.ciliata;* 4. *E. foemina;* 5. *E. pachyclada*

♀*: female;* ♂: male; //: as previous
